# Supplementary material for: Matrix stiffness controls lymphatic vessel formation through regulation of a GATA2-dependent transcriptional program
Source: Nat Commun. 2018 Apr 17;9:1511. doi: 10.1038/s41467-018-03959-6 (PMC5904183; doi:10.1038/s41467-018-03959-6)
Supplement: Supplementary file 1 — Supplementary Information [file 41467_2018_3959_MOESM1_ESM.pdf]

Supplementary Information

Matrix stiffness controls lymphatic vessel formation through regulation of a GATA2-dependent transcriptional program

Frye et al.

Supplementary Figures

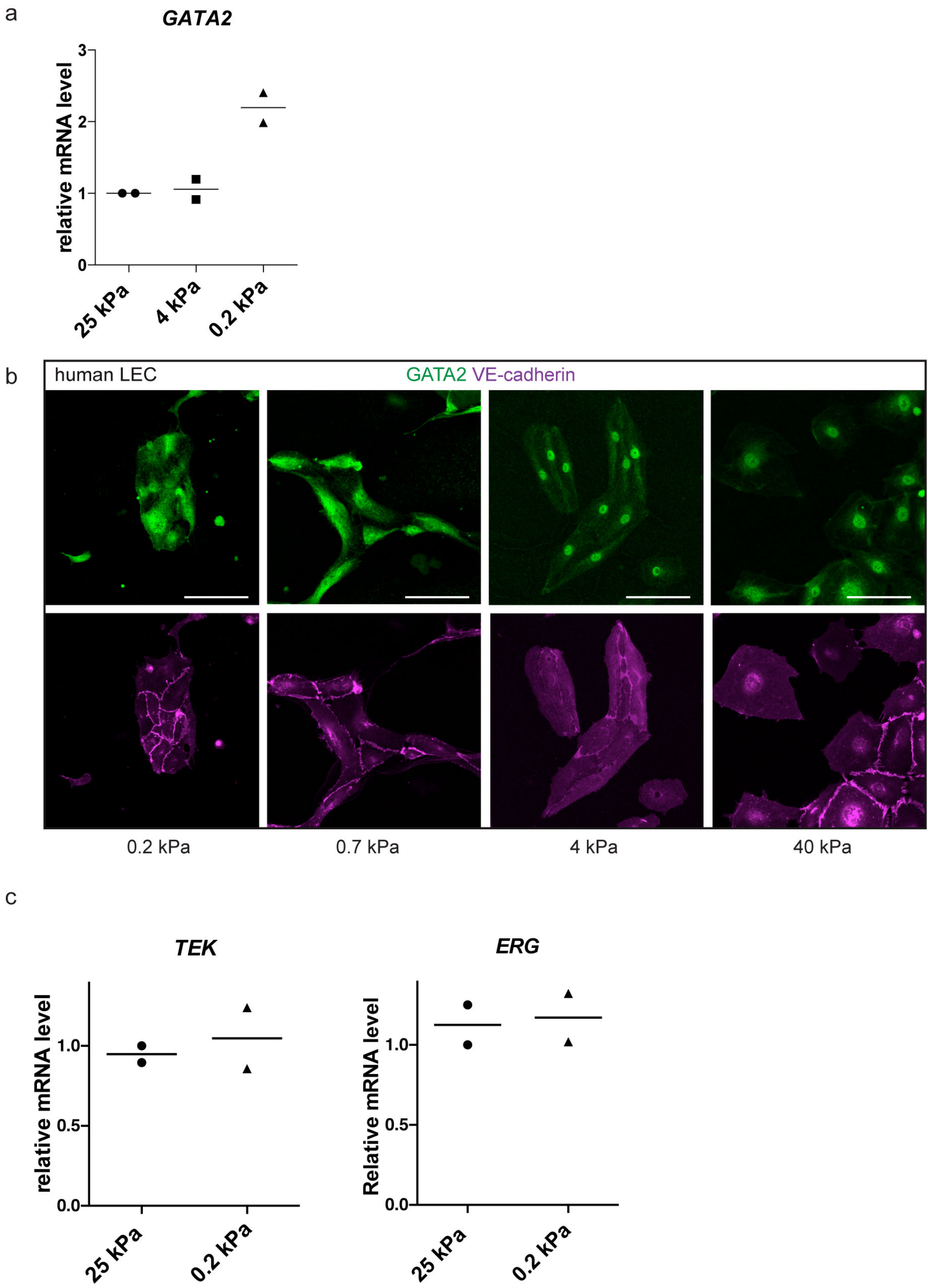

Supplementary Figure 1. GATA2 localization in matrices of different stiffness. (**a**) qRT-PCR analysis of *GATA2* in human LECs grown on matrix of 0.2 kPa, 4 kPa or 25 kPa stiffness. Horizontal lines represent mean (n=2 experiments). (**b**) Immunofluorescence of human LECs grown on matrices of indicated stiffness using antibodies against GATA2 (green) and VE-cadherin (magenta). Note nuclear localization of GATA2 at 4 kPa, which is equivalent to the stiffness of the embryonic cardinal vein (see Fig. 1e). (**c**) qRT-PCR analysis of endothelial markers *TEK* and *ERG* in human LECs grown on stiff (25 kPa) or soft (0.2 kPa) matrix. Horizontal lines represent mean (n=2). Neither endothelial marker is regulated by matrix stiffness. Scale bars: 100 µm (**b**).

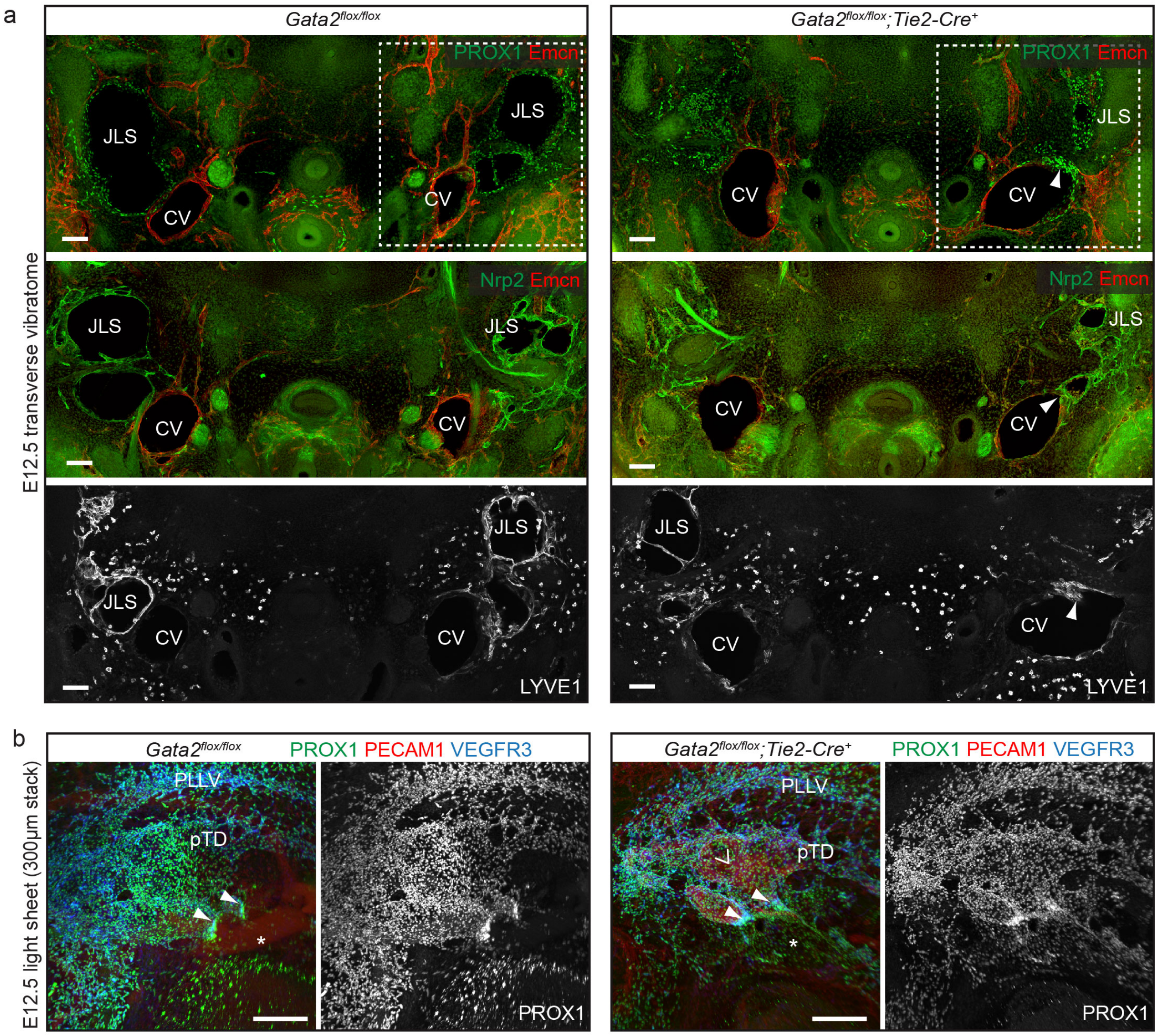

Supplementary Figure 2. Characterization of the vascular phenotype in *Gata2^{flox};Tie2-Cre* embryos. (**a**) Immunofluorescence of transverse vibratome sections of E12.5 embryos using indicated antibodies. Note smaller jugular lymph sac (JLS) and the presence of PROX1^{+}Nrp2^{high}LYVE1^{+}Emcn^{-} LECs (arrowheads) within the cardinal vein (CV) in the mutant embryo. Boxed areas in the upper panels are shown in Fig. 3e. (**b**) Maximum intensity projections of 300 µm stacks of E12.5 embryos (sagittal view) stained whole-mount for indicated proteins and imaged using light sheet microscopy. Single channel images for PROX1 staining are shown. Arrowheads point to lymphovenous valves that are abnormal in the mutant embryos. Asterisks indicate CV that contains PROX1^{+} cells in the mutant but not in the control. Open arrowhead shows autofluorescence from red blood cells inside the pTD in the mutant only. PLLV, peripheral longitudinal lymphatic vessel; pTD, primordial thoracic duct. Scale bars: 100 µm (**a**), 100 µm (**b**).

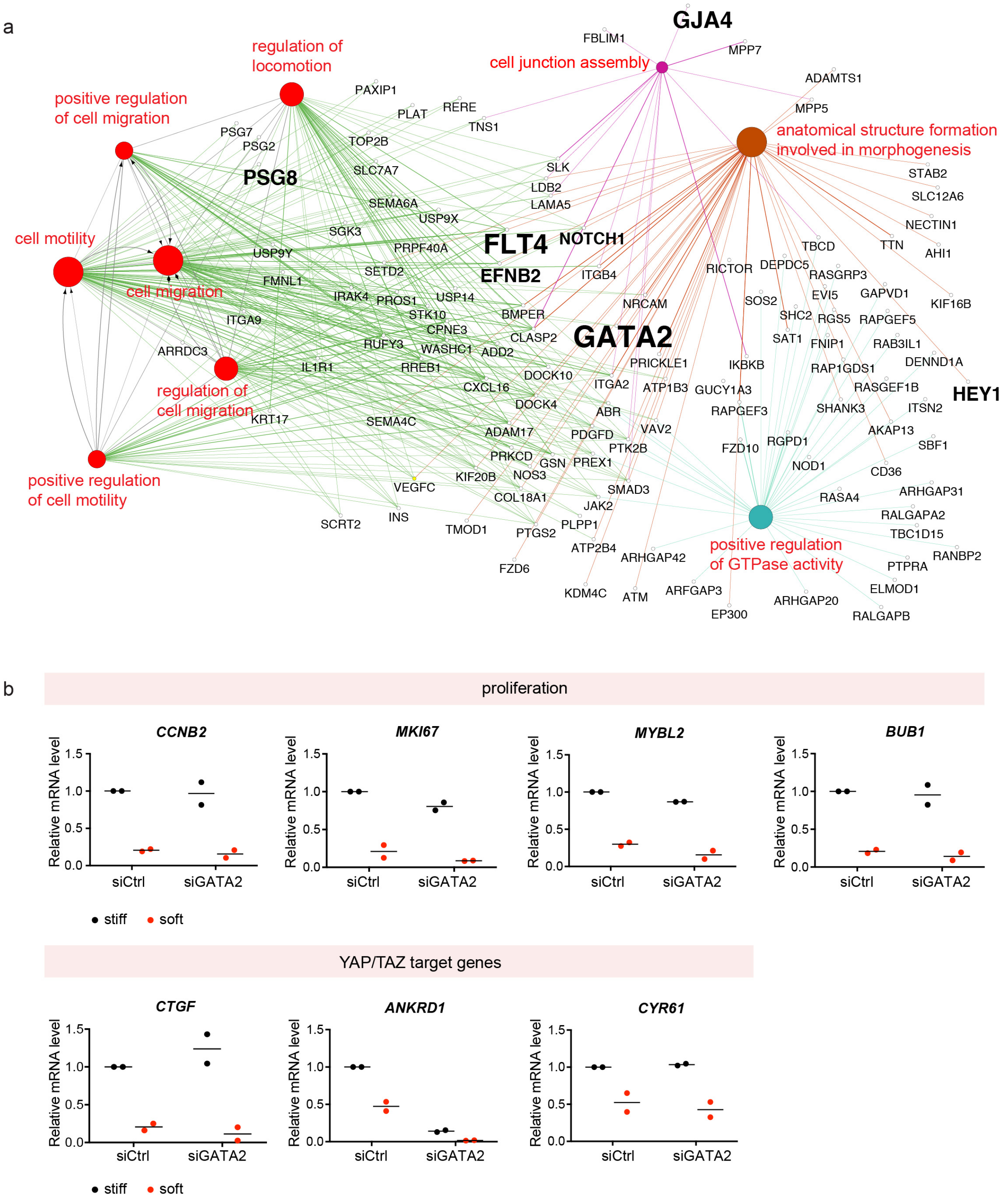

Supplementary Figure 3. Soft matrix induced (GATA2-regulated) biological processes and genes in LECs. (**a**) Visualization of biological process GO annotations and associated GATA2-regulated genes that were increased in LECs grown on soft (0.2 kPa) in comparison to stiff (25 kPa) matrix. Same colors of nodes indicate close relation of GO annotations. Relative size of nodes correlates with the number of regulated genes annotated to the specific GO term. Bold font indicates genes verified by qRT-PCR. *FLT4* and *GATA2* are verified *in vivo*. (**b**) qRT-PCR analysis showing soft matrix-induced downregulation of markers of proliferation (*CCNB2, MKI67, MYBL2, BUB1*) and YAP/TAZ target genes (*CTGF, ANKRD1, CYR61*) in human LECs both in the presence (siCtrl) and absence (siGATA2) of GATA2.

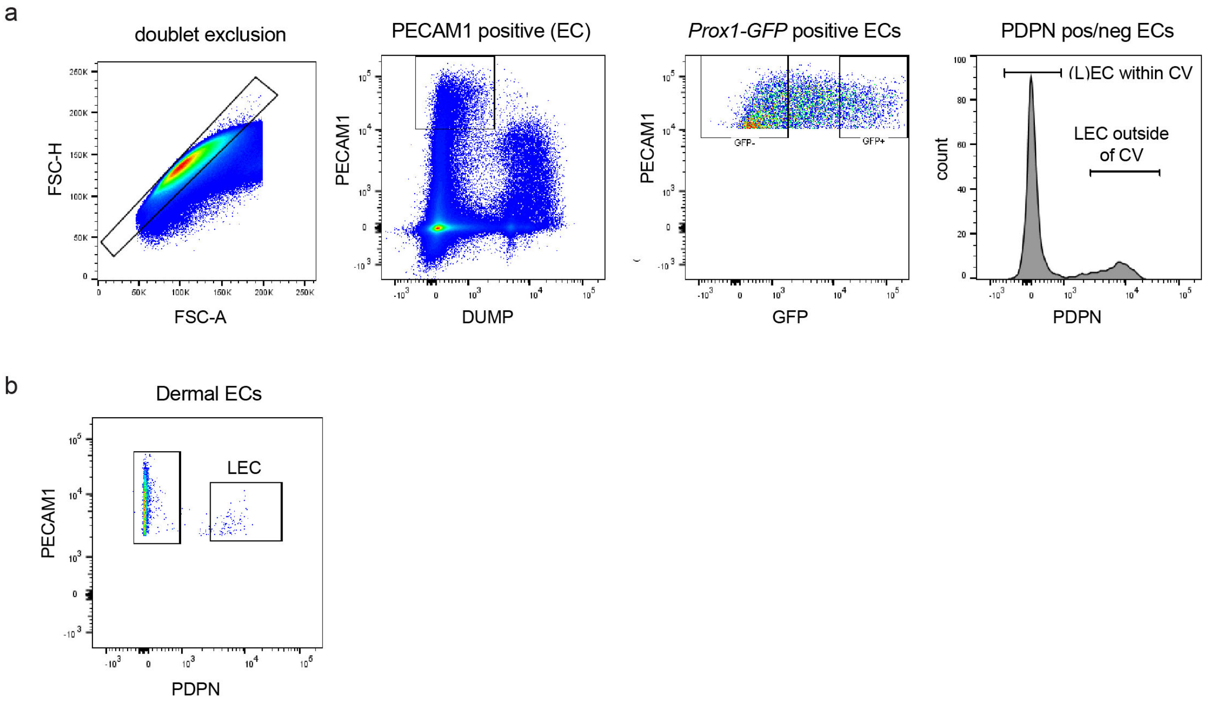

Supplementary Figure 4. Sorting of embryonic ECs. Gating scheme for the isolation of (**a**) ECs within and outside of the CV of E11 embryos and (**b**) dermal LECs from E15.5 *Gata2^{flox};Vegfr3-CreER^{T2}* mouse skin by flow cytometry. Doublet exclusion and selection of a PECAM1 positive population was performed as shown in (**a**).

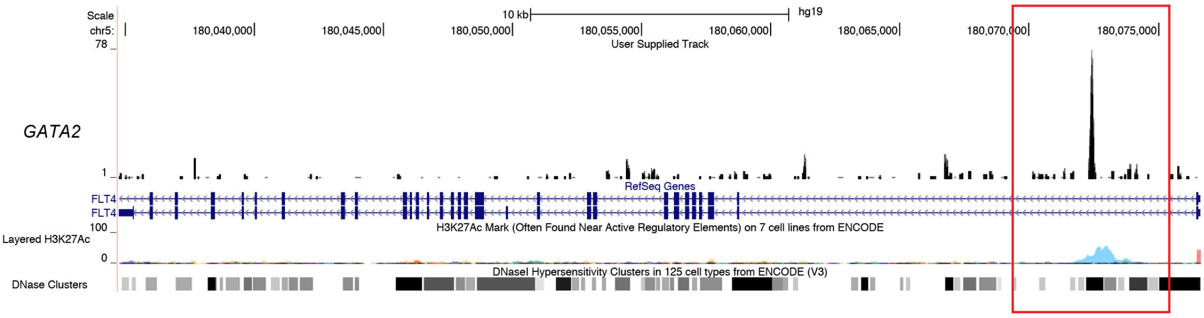

Supplementary Figure 5. Occupancy of chromatin at the *FLT4* (encoding VEGFR3) locus as viewed in UCSC Human Genome Browser (http://genome.ucsc.edu/). GATA2 ChIP-seq profile demonstrating binding at the first intron of the *FLT4* locus in human dermal LECs. Peak coordinates are chr5:180,072,303-180,072,606 (hg19). Co-occupancy with H3K27Ac and sites of DNase hypersensitivity, both marks of active enhancer elements, are shown in ENCODE tracks (HUVEC, light blue peaks). Boxed area is magnified in Fig. 5d.

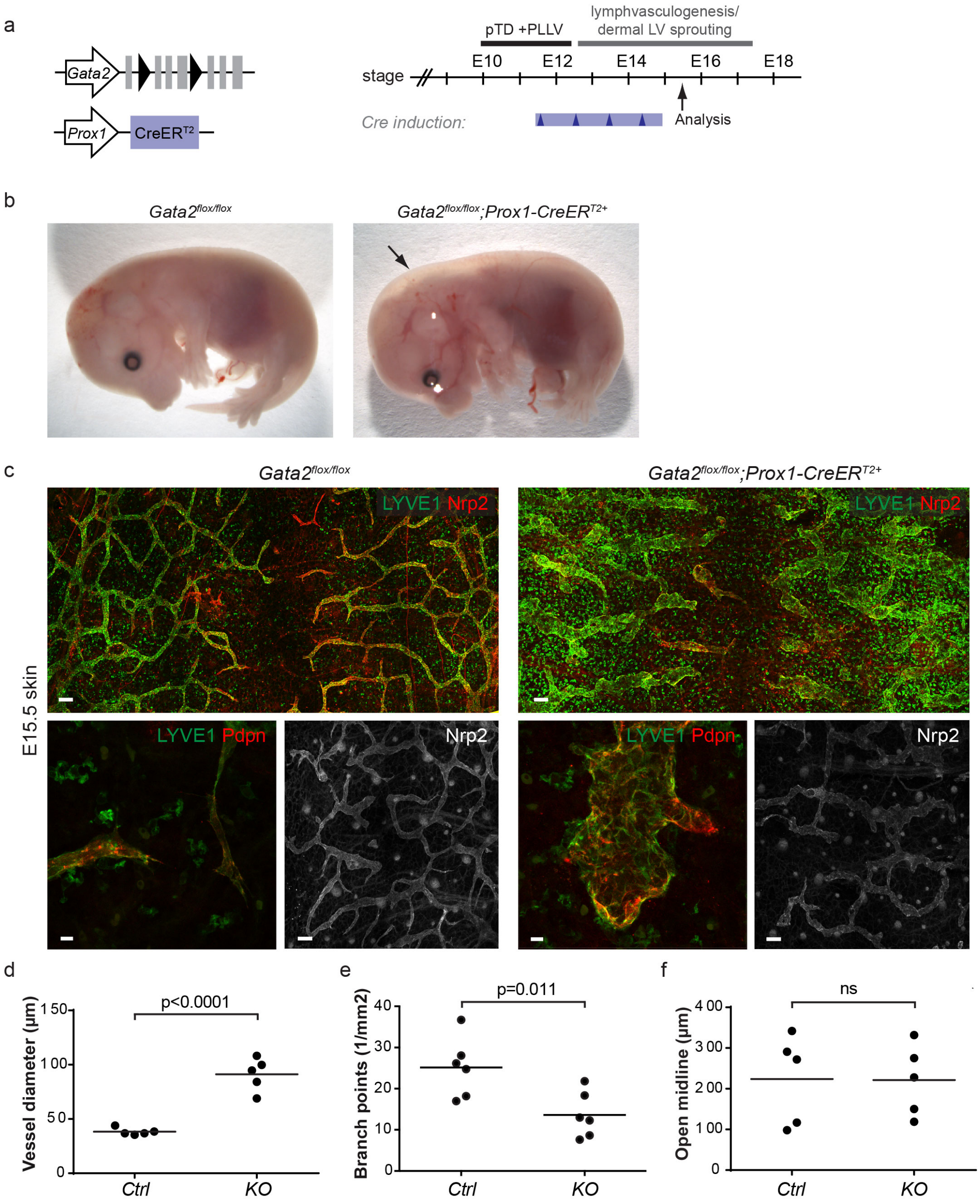

Supplementary Figure 6. Characterization of the vascular phenotype in *Gata2*^{flox}*;Prox1-CreER*^{T2} embryos. (**a**) Schematic of the genetic constructs, 4-OHT treatments (blue arrowheads; blue bars represent expected time frame of 4-OHT activity) and analyzed embryonic stage. The time frames for pTD+PLLV and dermal lymphatic vessel formation are indicated. (**b**) Subcutaneous edema in the *Gata2* mutant embryo (arrow). (**c**) Whole-mount immunofluorescence of E15.5 upper thoracic dorsal skin using indicated antibodies. Note the enlarged lymphatic vessels in the mutant. (**d-f**) Quantification of lymphatic vessel diameter (**d**), branch points (**e**) and the distance between contralateral sprouts (open midline; **f**) in E15.5 dorsal skin of *Gata2* mutant (KO) and littermate control (Ctrl) embryos. Horizontal lines represent mean (n=5 embryos in **d**, **f**; n=6 embryos in **e**). p value, Student's t-test. Scale bars: 100 µm (**c**; except for LYVE1/PDPN staining: 10 µm).

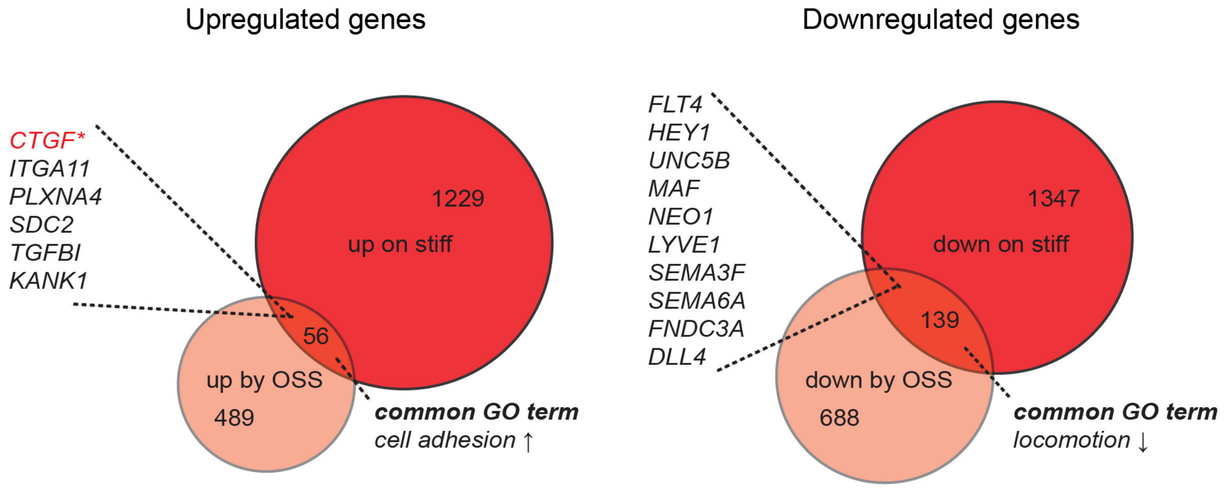

Supplementary Figure 7. General mechanoresponses in LECs. Area-proportional Venn diagrams of general mechanoresponsive genes in LECs grown on stiff in comparison to soft matrix, and LECs subjected to oscillatory flow as opposed to grown without flow[1]. The regulation of the YAP/TAZ target gene *CTGF* (red asterisk) is an indicator of mechanosignaling. Common TOP genes and selected GO term are listed.

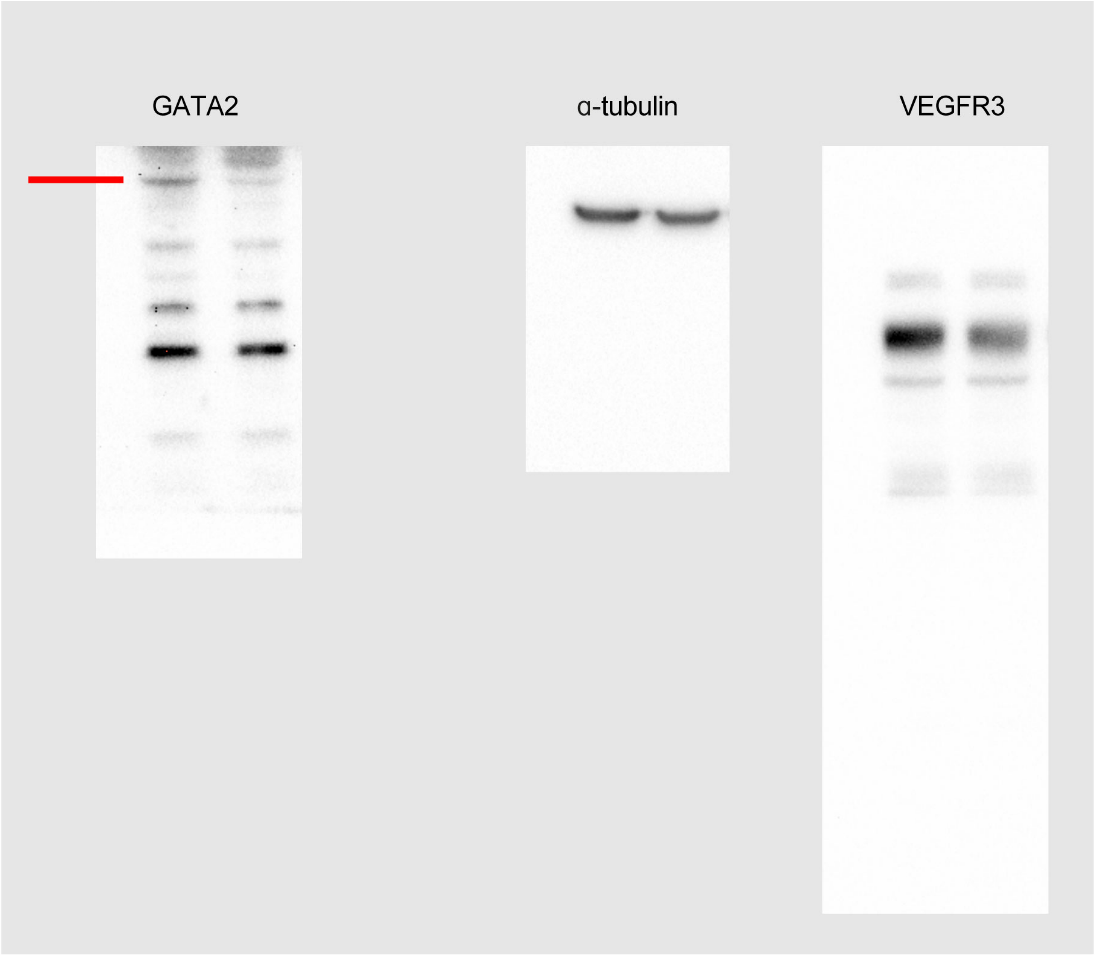

Supplementary Figure 8. Uncropped Western blots for Fig. 5e. Specific GATA2 band is indicated with a red band.

Supplementary References

1. Sabine, A. *et al.* Mechanotransduction, PROX1, and FOXC2 cooperate to control connexin37 and calcineurin during lymphatic-valve formation. *Dev. Cell* **22,** 430–445 (2012).
