# Supplementary material for: Matrix stiffness controls lymphatic vessel formation through regulation of a GATA2-dependent transcriptional program
Source: Nat Commun. 2018 Apr 17;9:1511. doi: 10.1038/s41467-018-03959-6 (PMC5904183; doi:10.1038/s41467-018-03959-6)
Supplement: Supplementary file 3 — Description of Additional Supplementary Files [file 41467_2018_3959_MOESM3_ESM.pdf]

Description of Additional Supplementary Files

File Name: Supplementary Movie 1.

Description: Volume reconstruction of whole-mount immunostained Gata2flox/flox embryo at E12.5. Movie showing the three-dimensional digital reconstruction of a Gata2flox/flox;Tie2-Cre- embryo, immunostained for PECAM1 (red), PROX1 (green) and VEGFR3 (blue). The specimen was imaged using a LaVision Ultamicrosope II light-sheet microscope, with a step size of 1 µm between sectional planes. The image stack was digitally rendered using the Voreen volume visualization framework.

File Name: Supplementary Movie 2.

Description: Volume reconstruction of whole-mount immunostained Gata2flox/flox;Tie2-Cre+ embryo at E12.5. Movie showing the three-dimensional reconstruction of a Gata2flox/flox;Tie2-Cre+ embryo, immunostained for PECAM1 (red), PROX1 (green) and VEGFR3 (blue). The specimen was imaged using a LaVision Ultamicrosope II light-sheet microscope, with a step size of 1 µm between sectional planes. The image stack was digitally rendered using the Voreen volume visualization framework. Note the reduced number of superficial sprouts towards the dorsal side of the embryo and blood inside the lymphatic vessels.

File Name: Supplementary Data 1

Description: Matrix stiffness regulated transcription factors. Differentially expressed transcription factors in LECs grown on stiff or soft matrix. The most updated database of transcription factors was exported for comparison [1]. From the 162 stiffness-regulated transcription factors, the potential core transcription factors controlling LEC response to matrix stiffness (highlighted in yellow, sheet 1) were predicted based on up- and downregulated genes with the online resource TFactS [2]. Additionally, the average fold change/log2 fold change/average expression (as average log2-intensity) of each transcription factor is shown in sheet 1. Separately, for each exon set ID, that passed the criteria for inclusion, the log2 fold change, average expression, t-test P-value from pairwise comparison of the 6 replicates and original raw log2-intensity data of all 6 replicates is shown for all upregulated transcription factor exon set IDs (sheet 2) and all downregulated transcription factor exon set IDs (sheet 3).

References for Supplementary Data 1:

[1] Schmeier, S., Alam, T., Essack, M. & Bajic, V. B. TcoF-DB v2: update of the database of human and mouse transcription co-factors and transcription factor interactions. Nucleic Acids Res. 45, D145–D150 (2017).

[2] Essaghir, A. et al. Transcription factor regulation can be accurately predicted from the presence of target gene signatures in microarray gene expression data. Nucleic Acids Res. 38, e120 (2010).
